# Identification of *Escherichia coli* strains using MALDI-TOF MS combined with long short-term memory neural networks

**DOI:** 10.18632/aging.205995

**Published:** 2024-06-29

**Authors:** Qiqi Mao, Xie Zhang, Zeping Xu, Ya Xiao, Yufei Song, Feng Xu

**Affiliations:** 1 Department of General Surgery, Li Huili Hospital Affiliated to Ningbo University, Ningbo 315040, China; 2 Department of Medicine and Pharmacy, Li Huili Hospital Affiliated to Ningbo University, Ningbo 315040, China; 3 School of Medicine, Ningbo University, Ningbo 315211, Zhejiang, China; 4 Department of Gastroenterology, Li Huili Hospital Affiliated to Ningbo University, Ningbo 315040, China

**Keywords:** matrix-assisted laser desorption ionization time-of-flight mass spectrometry, long short-term memory neural network, *Escherichia coli*, strain-level identification

## Abstract

The current study aims to develop a new technique for the precise identification of *Escherichia coli* strains, utilizing matrix-assisted laser desorption ionization time-of-flight mass spectrometry (MALDI-TOF MS) combined with a long short-term memory (LSTM) neural network. A total of 48 *Escherichia coli* strains were isolated and cultured on tryptic soy agar medium for 24 hours for the generation of MALDI-TOF MS spectra. Eight hundred MALDI-TOF MS spectra were obtained per strain, resulting in a database of 38,400 spectra. Fifty percent of the data was utilized for LSTM neural network training, with fine-tuned parameters for strain-level identification. The other half served as the test set to assess model performance. Traditional PCA dimension reduction of MALDI-TOF MS spectra indicated 47 out of 48 strains to be unclassifiable. In contrast, the LSTM neural network demonstrated remarkable efficacy. After 20 training epochs, the model achieved a loss value of 0.0524, an accuracy of 0.999, a precision of 0.985, and a recall of 0.982. When tested on the unseen data, the model attained an overall accuracy of 92.24%. The integration of MALDI-TOF MS and LSTM neural network markedly enhances the identification of *Escherichia coli* strains. This innovative approach offers an effective and accurate tool for MALDI-TOF MS-based strain-level identification, thus expanding the analytical capabilities of microbial diagnostics.

## INTRODUCTION

Matrix-assisted laser desorption/ionization time of flight mass spectrometry (MALDI-TOF MS) has become an invaluable tool in the rapid identification of microbial species. This technology employs laser energy to enable sample desorption and ionization prior to analysis in a time-of-flight mass spectrometer, determining the sample’s precise molecular weights [1–3]. MALDI-TOF MS focuses on whole bacterial proteins, yielding a characteristic “protein fingerprint.” These fingerprints are predominantly composed of highly-expressed, conserved ribosomal proteins, providing a reliable means for microbial species-level identification [4–6].

Despite its efficacy in rapid and accurate microbial identification, limitations of MALDI-TOF MS currently exist at the species level, owing to its reliance on microbial proteins [7, 8]. Strains within the same species exhibiting high protein expression similarity often remain undifferentiated. Advances in deep learning algorithms, especially long short-term memory (LSTM) neural networks, present a solution to this limitation. LSTMs are noted for their ability to manage long-term information through a network of input, forget, and output gates, enabling them to identify subtle variations in complex data sequences [9–11].

*Escherichia coli* is an exemplary subject for extending MALDI-TOF MS applications to strain-level identification. *Escherichia coli* strains function both as a harmless component of human flora and as a clinical pathogen. Strain-level identification is essential for tracing the origin of nosocomial infections and reducing associated risks. Recognizing this pressing need and the limitations of existing technologies, this study seeks to explore the utility of integrating MALDI-TOF MS with LSTM neural networks for strain-level identification of *Escherichia coli*. This exploration aims to establish a novel method that advances the analytical capabilities of MALDI-TOF MS, particularly in microbial strain-level diagnostics.

This introduction provides a foundation for the research by first examining the current state of MALDI-TOF MS technology, emphasizing its limitations, and then exploring the potential of LSTM neural networks to overcome these limitations [12, 13]. It also emphasizes the clinical importance of strain-level identification, especially for *Escherichia coli*, thereby establishing the relevance and significance of this study.

## MATERIALS AND METHODS

### Material and chemicals

In this study, 48 strains of *Escherichia coli* were extracted and purified from clinical biological samples. All isolates underwent biochemical testing and were subsequently confirmed as *Escherichia coli* via 16S rRNA gene sequencing. Tryptic Soy Agar (TSA) medium, sourced from Merck Millipore, Germany, was utilized for bacterial culture for MALDI-TOF MS analysis, α-Cyano-4-hydroxycinnamic acid (CHCA) served as the matrix and was obtained from Sigma-Aldrich, USA. The key instruments included a 4800 Plus MALDI-TOF MS mass spectrometer from Absciex, USA, and a DRP-9272 electric thermostatic microbial incubator provided by Shanghai Senxin Experimental Instrument Co., Ltd.

### MALDI-TOF MS analysis

Colonies of each bacterial strain, cultured over a 24-hour period, were prepared for analysis. A portion of the colony biomass was spread across assigned target sites on the MALDI plate. Subsequently, one microlitre of α-Cyano-4-hydroxycinnamic acid (CHCA) matrix solution was applied onto each sample spot. Afterward, the plate was left to air-dry, enabling matrix-sample co-crystallization. The prepared MALDI plate was then inserted into the MALDI-TOF MS instrument set to linear scanning mode. Laser intensity was adjusted to 3500 units, and the mass-to-charge (m/z) scanning range was established from 0 to 12,000 Da. For each bacterial strain, a total of 40 sample points were analyzed on the plate. From each point, 20 individual spectra were obtained, accumulating in a composite dataset of 800 spectra per strain. The signal-to-noise ratio for the most intense peak in each spectrum needed to exceed 10 for the data to be deemed valid. Additionally, intra-strain spectral variability was evaluated using Hotelling’s T^2^ statistical test, with an allowable variance of no more than 5%.

### Preparation of the dataset for MALDI-TOF MS spectral analysis

A comprehensive spectral database was created from the 38,400 acquired MALDI-TOF MS spectra, each meticulously categorized according to their originating strains. Each entry in the database represents a unique spectral signature. For each bacterial strain in the database, the dataset is divided into two mutually exclusive subsets. Specifically, 50% of the individual spectra for each strain are chosen using a stochastic sampling algorithm to form the training set. The remaining 50% comprise the test set. Before this division, all spectra undergo a quality control check to ensure compliance with pre-defined data quality standards, including, but not limited to, signal-to-noise ratios and Hotelling’s T^2^ statistical thresholds.

### LSTM network model architecture and training protocol

The LSTM model is built using the Tensorflow v2.0 framework. It comprises an LSTM layer, a fully connected layer, and a Dropout layer, with parameters set at 128, 64, and 0.3, respectively. Layers are sequentially connected. Details such as activation functions and output sizes are provided in Table 1. The training loss function is categorical cross-entropy, the optimizer is Adam, and 80% of the spectra in the training set are randomly chosen for training, with the remaining 20% used for cross-validation. The maximum training duration is 20 epochs. Model training results are assessed using precision, accuracy, and recall metrics. The calculation formulae are presented in formula [1–3], where TP represents the positive samples correctly predicted by the model, TN the negative samples correctly predicted, FP the negative samples incorrectly predicted as positive, and FN the positive samples incorrectly predicted as negative.

**Table 1:** Structure, activation function, and parameters of the LSTM model.

**Layer**	**Layer (type)**	**Activation function**	**Output size**	**Number of parameters**	**Total parameters**
1	Lstm (LSTM)	Relu	(None, 128)	1348608	1359984
2	Dense1 (Dense)	Relu	(None, 64)	8256	
3	Dropout (Dropout)		(None, 64)	0	
4	Dense (Dense)	Softmax	(None, 48)	3120	


$$Precision=\frac{TP}{TP+FP}\;\;\;\;(1)$$

$$Accuracy=\frac{TP+TN}{TP+TN+FP+FN}\;\;\;\;(2)$$

$$Recall=\frac{TP}{TP+FN}\;\;\;\;(3)$$

### Model evaluation metrics and analysis

The model’s predictive performance is evaluated using a detailed confusion matrix. This matrix classifies multi-class classification outcomes into categories of True Positives (TP), False Positives (FP), True Negatives (TN), and False Negatives (FN), each quantified with absolute numerical values. A crucial metric for model assessment is the Comprehensive Recognition Rate, defined as the ratio of accurately classified samples to the total number of samples in the test dataset. Mathematically, this rate is expressed as:

Comprehensive Recognition Rate = Total Number of Test Samples/Number of Correctly Classified Samples.

### Availability of data and material

The datasets generated and/or analyzed during the current study are available in the SimTK repository (https://simtk.org/plugins/datashare/?group_id=2836). The 16S rRNA sequencing data are also available in the SimTK repository (https://simtk.org/plugins/datashare/?group_id=2836).

## RESULTS

### Strain MALDI-TOF MS spectrum database

**Figure 1:**
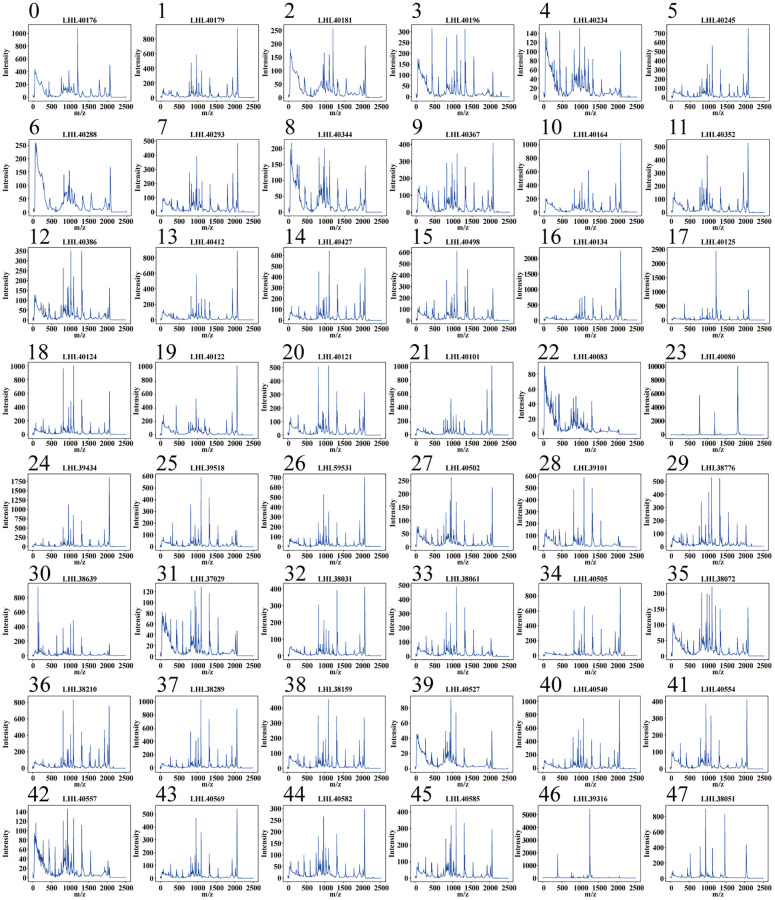
Typical MALDI-TOF MS patterns for 48 *Escherichia coli* strains.

**Figure 2:**
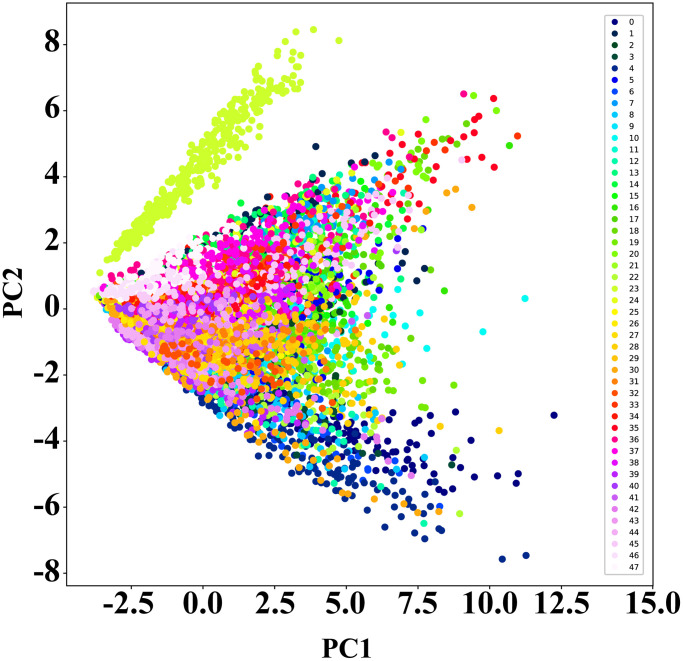
**PCA dimensionality reduction results for *Escherichia coli* strain MALDI-TOF MS spectra.** The numbers 0–47 represent the 48 *Escherichia coli* strains used in the study. The isolated cluster of points in the upper left corner corresponds to strain No. 23 LHL40080.

The constructed database includes a total of 38,400 MALDI-TOF MS spectra, equally distributed among 48 distinct strains of *Escherichia coli*. Each strain contributes 800 individual spectra, providing a balanced dataset for further analysis. Figure 1 shows the typical MALDI-TOF MS spectra for the 48 *Escherichia coli* strains. Significant peaks, specific to each strain, are primarily noted in the m/z range of 2000 to 10,000. Principal Component Analysis (PCA) is utilized for dimensionality reduction, as shown in Figure 2. Interestingly, the primary component for the spectra of strain LHL40080 (Strain No. 23) appears in the upper left quadrant of the scatter plot, demonstrating distinct data separability from the other 47 strains. However, the principal components for the spectra of the other 47 strains overlap significantly, making them challenging to distinguish. Upon closer examination, it was noted that the MALDI-TOF MS spectra for strain No. 23 differed markedly from those of other strains. This differentiation is presumed to result from subspecies-level variation in this particular strain. Further research is needed to verify this hypothesis.

### Model training and performance metrics

**Figure 3:**
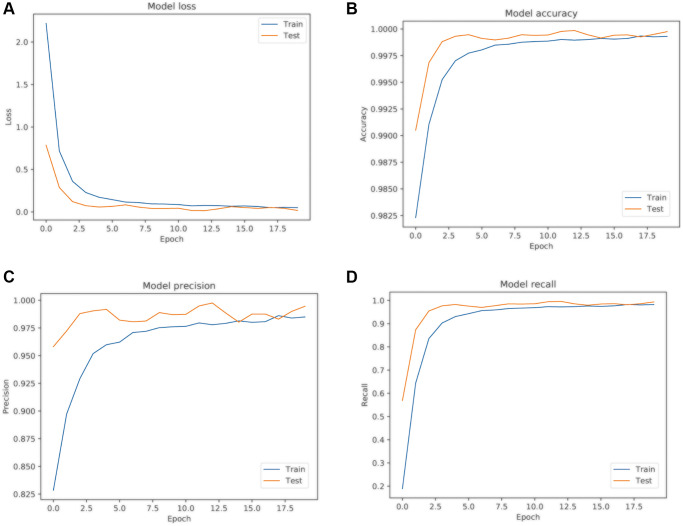
**LSTM model training results.** (**A**) Model loss curve; (**B**) Model accuracy curve; (**C**) Model precision curve; (**D**) Model recall curve. Blue represents the training sample curve, and yellow represents the test sample curve.

After completing 20 training epochs, as illustrated in Figure 3, our Long Short-Term Memory (LSTM) model demonstrated exemplary performance metrics, validating its effectiveness for the intended application. Specifically, the model recorded a remarkably low loss value of 0.0524, reflecting optimal minimization of prediction errors. This was paired with an exceptional accuracy rate of 0.999, highlighting the model’s near-flawless class label predictions. Additionally, a precision metric of 0.985 and a recall score of 0.982 together indicate the model’s high specificity and sensitivity, affirming its robustness in minimizing false positives and false negatives. These combined metrics underscore the model’s overall predictive prowess and reliability.

### Model evaluation

**Figure 4:**
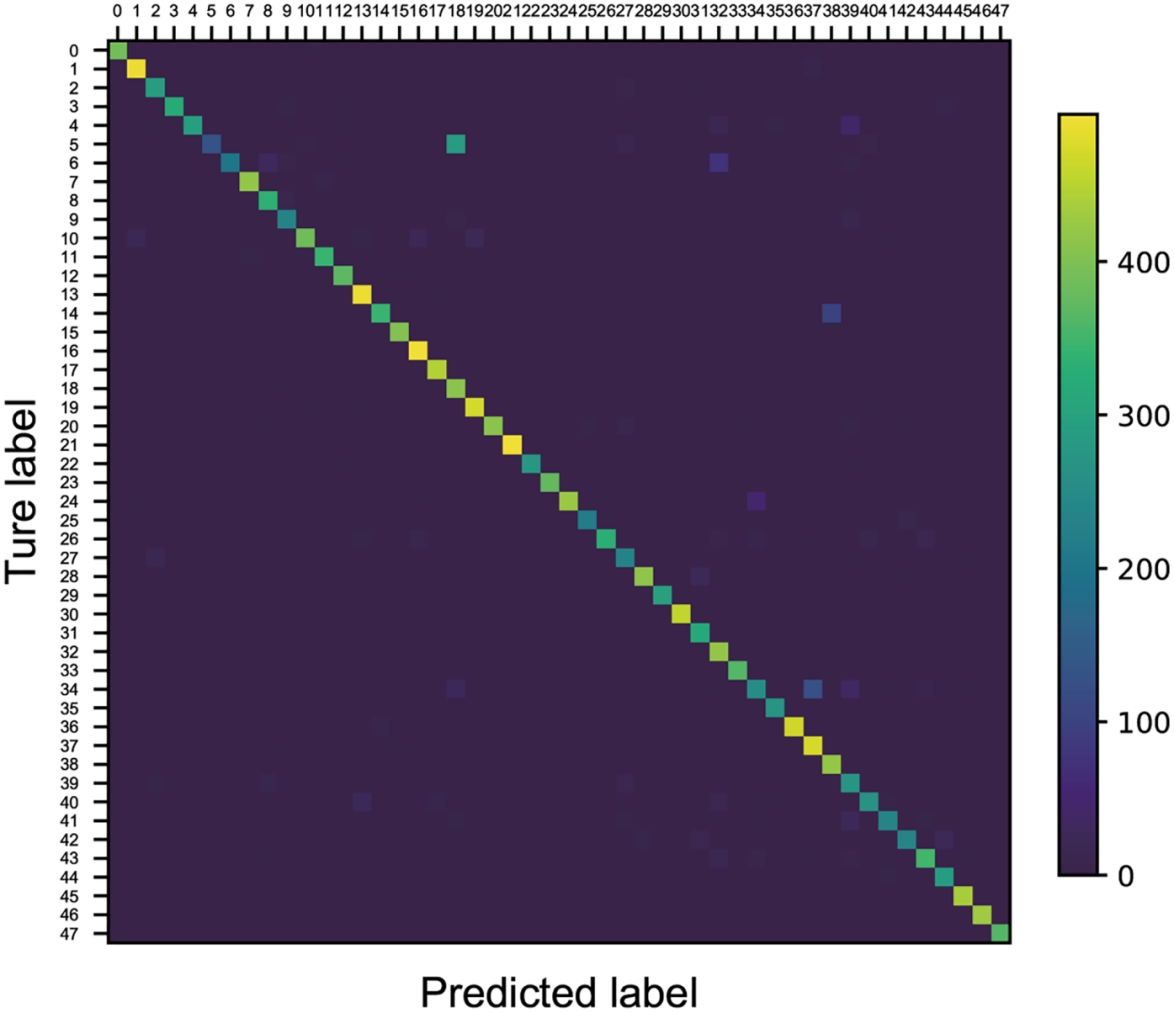
**Confusion matrix for model evaluation.** The numbers 0–47 refer to the 48 *Escherichia coli* strains used in the study.

The confusion matrix presented in Figure 4 provides crucial insights into classification discrepancies among various strains. For example, strain No. 5 was predominantly misclassified as strain No. 18 at a 71% rate (284 out of 400 samples), strain No. 14 was misidentified as strain No. 38 in 25.75% of cases (103 out of 400 samples), and 31.25% of samples from strain No. 34 were misclassified as strain No. 37 (125 out of 400 samples). Despite these specific instances of misclassification, the model exhibited robust performance for the remaining strains, achieving an identification accuracy exceeding 90%. The overall identification accuracy across all 48 strains reached a commendable 92.24%.

## DISCUSSION

In the realm of microbial diagnostics, traditional MALDI-TOF MS technology has proven to be a rapid and accurate tool by generating specific bacterial fingerprint spectra through the analysis of microbial cellular proteins and peptides [14, 15]. However, this method encounters challenges in differentiating at the subspecies level or among similar microorganisms [16]. Traditional algorithms, while providing statistical validity to matching scores using probabilistic frameworks, are limited in distinguishing closely related strains due to the randomness in the MALDI-TOF MS sampling process [17, 18].

In our newly developed method, by incorporating LSTM neural networks, we are able to overcome these limitations. The unique architecture of LSTMs enhances control over information flow and improves data processing capabilities, particularly in handling long-term dependencies related to time series [19]. The LSTM networks can perform finer analysis of subtle differences within complex biological samples, identifying specific spectral peaks or patterns associated with virulence factors, toxins, or other biomarkers. This ability is crucial for differentiating microbes that have minor variations in their biomarkers [20].

Additionally, compared to traditional methods, the combined MALDI-TOF MS and LSTM approach is more efficient in handling large datasets, as LSTM networks are designed to manage extensive datasets, offering quicker processing times and more efficient data handling than conventional statistical methods. This is particularly useful for the voluminous data often generated by MALDI-TOF MS. The attributes of LSTM networks make them an ideal choice for predictive modeling in the complex biological systems analyzed by MALDI-TOF MS, leading to the development of superior prognostic and diagnostic tools.

In this study, 800 maps of each strain were gathered in batches to form a training dataset, preserving the unique characteristics of all maps. Utilizing an LSTM neural network, feature extraction and classification training were conducted on 2505-dimensional atlas data [21, 22]. Retaining the feedback mechanism of the recurrent neural network (RNN), the LSTM enhances data processing by introducing gating units such as forgetting gates, input gates, and output gates. This approach enables better control over information transmission and addresses issues like RNN gradient disappearance and difficulty in capturing long-term sequence dependencies [20, 23–25]. Consequently, inputting the 2505-length map data into the LSTM neural network results in effective feature extraction and classification. Through training on 19,200 images, the LSTM model learned the characteristics of the MALDI-TOF MS spectra at the strain level, achieving the identification of 48 *Escherichia coli* strains with a comprehensive accuracy of 92.24%. These results demonstrate that MALDI-TOF MS provides high-resolution mass spectrometry data, enabling precise and accurate analysis of complex biological samples. When integrated with LSTM neural networks, this precision is further enhanced as the LSTM can efficiently process and interpret the intricate mass spectrometry data. MALDI-TOF MS often generates large datasets. LSTM neural networks are well-suited for managing such large datasets efficiently, offering quicker processing times and more efficient data handling than traditional statistical methods. LSTM networks’ ability to learn from and remember long sequences makes them ideal for predictive modeling in complex biological systems analyzed by MALDI-TOF MS, leading to better prognostic and diagnostic tools.

While our study marks a significant advancement in the field of microbial diagnostics, we acknowledge certain limitations that must be considered. One of the most notable limitations is the absence of a direct experimental comparison between our novel method and existing microbial identification techniques. Such a comparison could have provided a more robust foundation for validating our approach. Additionally, the lack of a definitive determination method, like Multi-Locus Sequence Typing (MLST) or genome MLST (gMLST), to confirm whether certain strains, such as strain 23, are subspecies of *E. coli*, is a notable limitation of our study. Despite these constraints, the integration of LSTM neural networks with MALDI-TOF MS technology represents a significant leap forward in microbial diagnostics.

Moreover, our study delves deeper into the potential of this methodology in identifying specific markers for the accurate discrimination of *E. coli* categories and its applicability in identifying pathogenic strains. This exploration not only highlights the novel contributions of the present study but also opens new avenues for future research in the field of microbial diagnostics. It suggests the possibility of developing more refined tools for microbial identification that could significantly impact clinical diagnostics and public health.
